# Electrolyte derangements in critically ill children receiving balanced versus unbalanced crystalloid fluid resuscitation

**DOI:** 10.1186/s12882-022-03009-w

**Published:** 2022-12-06

**Authors:** Natalja L. Stanski, Katja M. Gist, Kaci Pickett, John T. Brinton, Jennifer Sadlowski, Hector R. Wong, Peter Mourani, Danielle E. Soranno, Jessica Kendrick, Erin K. Stenson

**Affiliations:** 1 Division of Critical Care Medicine, Cincinnati Children’s Hospital Medical Center, Cincinnati, OH USA; 2 Department of Pediatrics, University of Cincinnati College of Medicine, Cincinnati, OH USA; 3 Division of Cardiology, Cincinnati Children’s Hospital Medical Center, Cincinnati, OH USA; 4 Department of Biostatistics and Informatics, University of Colorado Anschutz Medical Campus, Aurora, CO USA; 5 Department of Pediatrics, University of Colorado School of Medicine, Aurora, CO USA; 6 Research Informatics, Children’s Hospital Colorado, Aurora, CO USA; 7 Department of Pediatrics, University of Arkansas for Medical Sciences College of Medicine, Little Rock, AR USA; 8 Division of Critical Care Medicine, Arkansas Children’s Hospital, Little Rock, AR USA; 9 Section of Nephrology, University of Colorado School of Medicine and Children’s Hospital Colorado, Aurora, CO USA; 10 Division of Renal Disease and Hypertension, University of Colorado Anschutz Medical Campus, Aurora, CO USA; 11 Section of Critical Care, University of Colorado School of Medicine and Children’s Hospital Colorado, 13121 E 17th Avenue, MS8414, Aurora, CO 80045 USA

**Keywords:** Lactated Ringers, Sodium chloride, Hyperkalemia, Hyponatremia, Acidosis

## Abstract

**Background:**

Adult studies have demonstrated potential harm from resuscitation with 0.9% sodium chloride (0.9%NaCl), resulting in increased utilization of balanced crystalloids like lactated ringers (LR). The sodium and potassium content of LR has resulted in theoretical safety concerns, although limited data exists in pediatrics. We hypothesized that use of LR for resuscitation would not be associated with increased electrolyte derangements compared to 0.9%NaCl.

**Methods:**

A prospective, observational cohort study of critically ill children who received ≥ 20 ml/kg of fluid resuscitation and were admitted to two pediatric intensive care units from November 2017 to February 2020. Fluid groups included patients who received > 75% of fluids from 0.9%NaCl, > 75% of fluids from LR, and a mixed group. The primary outcome was incidence of electrolyte derangements (sodium, chloride, potassium) and acidosis.

**Results:**

Among 559 patients, 297 (53%) received predominantly 0.9%NaCl, 74 (13%) received predominantly LR, and 188 (34%) received a mixture. Extreme hyperkalemia (potassium ≥ 6 mmol/L) was more common in 0.9%NaCl group (5.8%) compared to LR group (0%), p 0.05. Extreme acidosis (pH > 7.1) was more common in 0.9%NaCl group (11%) compared to LR group (1.6%), p 0.016.

**Conclusions:**

LR is associated with fewer electrolyte derangements compared to 0.9%NaCl. Prospective interventional trials are needed to validate these findings.

**Supplementary Information:**

The online version contains supplementary material available at 10.1186/s12882-022-03009-w.

## Background

Fluid resuscitation with crystalloid solutions is a mainstay of therapy in critically ill children [1]. The most frequently utilized crystalloid in both adult and pediatric patients is 0.9% sodium chloride (0.9%NaCl) [2], despite a growing body of evidence to suggest potential harm from its use, including higher rates of mortality, acute kidney injury (AKI), and electrolyte and acid–base disturbances [3–10]. The detrimental effects of resuscitation with 0.9%NaCl are hypothesized to be secondary to supraphysiologic amounts of chloride compared to plasma (154 mEq/L versus ~ 100 mEq/L) in unbalanced crystalloids [11]. As a result, the use of balanced crystalloids such as lactated ringers (LR) (110 mEq/L chloride) and plasma-lyte 148 (PL) (98 mEq/L chloride) is becoming increasingly more common in critically ill patients, including children. However, the lower concentrations of sodium (130 mEq/L in LR and 140 mEq/L in PL, compared to 154 mEq/L in 0.9%NaCl) and higher amounts of potassium (4 mEq/L in LR and 5 mEq/L in PL, compared to none in 0.9%NaCl) in these balanced crystalloid solutions have led to theoretical concerns for hyponatremia and hyperkalemia if used for resuscitation. While small adult studies [4, 12, 13] and studies in specific pediatric diseases [14, 15] have evaluated and dispelled some of these concerns, there remains a paucity of data regarding the association between balanced crystalloids and electrolyte derangements following resuscitation in heterogeneous populations of critically ill children.

The purpose of this study was to examine the incidence of hyperkalemia, hyponatremia, hyperchloremia, and acidosis in critically ill children who primarily received balanced crystalloids compared to those who received 0.9%NaCl for resuscitation. We hypothesized a priori that use of balanced crystalloids for fluid resuscitation would be associated with a lower incidence of electrolyte derangements and acidosis when compared to 0.9%NaCl.

## Methods

### Study design

We conducted a prospective, observational cohort study that evaluated the use of different types of resuscitative fluids (0.9% NaCl, LR, or mixed) and resultant electrolyte values among pediatric patients who were admitted to two large quaternary pediatric intensive care units (PICUs), Cincinnati Children’s Hospital Medical Center (CCHMC) and Children’s Hospital Colorado (CHCO), between November 2017 and February 2020. This study was approved by the institutional review boards at both institutions with a waiver of informed consent.

### Patient selection

All patients ≥ 1 month and < 18 years of age who were admitted to the PICU were assessed for eligibility. Inclusion criteria were the following: 1) receipt of at least one fluid bolus (≥ 20 mL/kg or 1 L if ≥ 50 kg) during the 12 h prior to PICU admission *or* within the first 24 h after PICU admission; 2) PICU length of stay (LOS) greater than 48 h; and 3) at least 2 measurements of pH with serum electrolyte values (sodium, chloride, potassium) within the first 7 days of PICU admission. Exclusion criteria included: 1) a diagnosis of chronic kidney disease, defined as Kidney Disease: Improving Global Outcomes (KDIGO) Criteria stage G2 (mildly decreased GFR ≥ 60 to < 90 ml/min/1.73m^2^) to stage G5 (kidney failure with GFR < 15 ml/min/1.73m^2^) [16]; 2) patients admitted with traumatic brain injury or pre-/post-operative from neurosurgical procedures, due to the use of hypertonic saline for treatment/prevention of cerebral edema and exposure to an extreme sodium and chloride load; and 3) patients who were transferred from facilities outside of the hospital care network, due to incomplete documentation of fluids administered. Patients with complex congenital heart disease were also excluded, as they are managed in a separate cardiac intensive care unit at each site.

### Data collection

All enrolled patients had daily clinical and laboratory data collected for up to 7 days (Day_0_ as the calendar day of PICU admission, through Day_7_), per standard clinical care until transfer out of the PICU, or death, whichever came first. Laboratory data included serum electrolytes (sodium, chloride and potassium) and pH. If there were multiple laboratory values on a given day, the highest potassium and chloride values, and the lowest sodium and pH values were recorded. Only non-hemolyzed samples were assessed which was determined via chart review and via coding of the EMR data extraction. Clinical data included the amount (indexed for body weight), type, and electrolyte content of fluid administered. Outcome data were tracked for 28 days after PICU admission. At CCHMC, all data was manually extracted from the electronic medical record (EMR) and entered into the REDCap database by 2 investigators (N.L.S., E.K.S.). At CHCO, data was exported to REDCap via an informatics query from the EMR or manually extracted (E.K.S.). A subset of informatics extracted data was randomly selected and manually verified (E.K.S.). Severity of illness was assessed on admission using the Pediatric Risk of Mortality III (PRISM-III) score [17].

### Definitions of fluid exposure groups

The total amount of fluid volume for each patient was calculated by adding any bolus fluids (denoted by “bolus” in the intake flowsheet of the electronic health record) received from 12 h preceding PICU admission up to 24 h after. Maintenance fluid was also included in this exposure group. Both bolus and maintenance fluid given within this time period were included in the overall fluid exposure calculations.

Based on the composition of fluids received, patients were divided into fluid exposure groups for comparison:

1. 0.9%NaCl group included patients who received ≥ 75% of fluid as 0.9%NaCl
2. LR group included patients who received ≥ 75% of fluid as LR or PL
3. Mixed group included patients who received a mixture of these fluid types but did not have ≥ 75% predominance of fluid type.

LR and PL were considered together due to their relatively infrequent use of PL across both centers. Total sodium and chloride loads were calculated based on the volume of fluid administered and the known concentration of sodium and chloride contained: 154 mEq/L of sodium and chloride in 0.9%NaCl, 130 mEq/L of sodium and 110 mEq/L of chloride in LR, and 140 mEq/L of sodium and 98 mEq/L of chloride in PL [18–20]. These electrolyte compositions are the same for both bolus fluid and maintenance fluids.

### Definitions of electrolyte derangements

Electrolyte values were initially considered as continuous variables, with median values for each group assessed and compared daily from Day_0_ through Day_2_. The incidence of electrolyte abnormalities was then assessed by defining each electrolyte derangement as a dichotomous variable for comparison, with cutoffs defined a priori based on the laboratory’s upper and lower limits of normal: hyponatremia defined as sodium < 135 mmol/L, hyperkalemia as potassium ≥ 5 mmol/L, hyperchloremia as chloride ≥ 110 mmol/L, and acidosis as pH < 7.3 (based on arterial, venous, or capillary blood gas). Patients were determined to have one of these electrolyte derangements if they were recorded as having at least a single measurement above or below these pre-defined cutoffs at any point from Day_0_ to Day_2_. Further post hoc analyses using more clinically significant cutoffs were also performed, including hyponatremia defined as ≤ 125 mmol/L and ≤ 130 mmol/L, hyperkalemia defined as ≥ 5.5 mmol/L and ≥ 6 mmol/L, hyperchloremia as ≥ 115 mmol/L and ≥ 120 mmol/L, and acidosis as pH ≤ 7.2 and ≤ 7.1. These cutoffs were defined based on previously published definitions of more profound derangements, and investigator consensus of thresholds likely to be relevant to patient care and require intervention [21–24].

### Outcomes

The primary outcome was the frequency of electrolyte derangements (specifically hyponatremia, hyperkalemia and hyperchloremia) and acidosis across the 3 fluid exposure groups. Outcomes data were also assessed and compared for each fluid exposure group, including PICU LOS, day 2–3 severe AKI (KDIGO stage 2–3), and 28-day mortality.

### Statistical analysis

Power and sample size were determined based on derangement of chloride as data exists across a variety of populations, with rates of hyperchloremia ranging from 10%-60% in prior studies [9, 25]. Sample size was estimated using a two-sided Fisher’s Exact test for a difference in proportions with an alpha = 0.05 significance level at 80% power. Sample size was estimated for the two-group comparison of hyperchloremia rates. We assumed a 20% rate of hyperchloremia and a 10% difference in hyperchloremia rates as a clinically important threshold and calculated that 484 patients were needed to detect a difference between groups.

The Shapiro–Wilk test determined non-normality of the daily electrolyte values. Plots also indicated a non-normal distribution and strong outliers. Daily electrolyte data were summarized as medians, interquartile ranges, frequencies, and percentages. The categorical exposure and the dichotomous outcome variables were defined as described above. Comparisons of clinical, demographic, and basic outcome variables between groups were performed with Kruskal–Wallis (KW), Fisher’s Exact, or Chi Square Tests, as appropriate. The KW test was used to compare daily electrolyte values which have a non-normal distribution and strong outliers. The test may perform better when the assumptions of the ANOVA are violated [26]. When overall tests were statistically significant, Bonferroni corrections were used for pairwise comparisons between the groups. A *p*-value of < 0.05 was considered statistically significant for all tests, except for measures repeated on multiple days and pairwise comparisons, for which a *p*-value of < 0.017 was used. Two sub-analyses comparing basic demographic, outcome, and fluid bolus selection data by site, as well as the impact of larger resuscitation volumes (> 60 ml/kg) were also performed. All statistical analyses were performed using SAS software 9.4 (SAS Institute Inc, Cary, North Carolina) and R software version 3.6.3, (R Foundation for Statistical Computing, Vienna, Austria, http://www.R-project.org/).

## Results

### Baseline characteristics

There were 559 patients included in this study (300 from CCHMC and 259 from CHCO). Three exposure groups were identified: 297 (53%) received at least 75% of fluids as 0.9%NaCl, 74 (13%) received at least 75% as LR, and 188 (34%) received a mixture of LR and 0.9%NaCl. Only two patients received PL and were included in the LR group. Table 1 summarizes clinical, demographic and outcomes characteristics according to fluid exposure group. There were no significant differences noted in age, gender, or admission weight between the three groups; however, patients in the LR and mixed groups had higher PRISM-III scores on admission (*p* = 0.04). Patients with a respiratory admission diagnosis were more likely to receive 0.9%NaCl for resuscitation (*p* = 0.043), while post-surgical and/or trauma patients were more likely to receive LR (*p* = 0.001). There were no significant differences in PICU LOS, incidence of day 2–3 severe AKI, or 28-day mortality across the three groups.

**Table 1:** Basic demographic, clinical and outcome data by fluid bolus exposure group

	**Overall**	**0.9%NaCl**	**LR**	**Mixed**	***p*-value**
**N (% cohort)**	559	297 (53)	74 (13)	188 (34)	
**Age, months**	44 (14,130)	37 (13,121)	60 (22,137)	62 (15,144)	0.06
**Gender, n (% male)**	311 (56)	172 (58)	44 (60)	95 (51)	0.22
**Weight, kg**	16 (9.8, 31)	14.5 (9.1, 30.4)	18.9 (10.5, 27.8)	18 (9.9, 32.9)	0.26
**PRISM III**	3 (0, 7)	3 (0,7)^a^	4 (0,8)^ab^	4 (2,8)^b^	0.04
**Admission Diagnosis, n (%)**					
Shock	143 (26)	80 (27)	12 (16)	51 (27)	0.14
Cardiovascular	3 (0.5)	2 (0.7)	1 (1.4)	0 (0)	0.36
Respiratory	318 (57)	183 (61)	40 (54)	95 (51)	0.043
Surgical/trauma	16 (2.9)	4 (1.4)	7 (9.5)	5 (2.7)	0.001
CNS	14 (2.5)	8 (2.7)	1 (1.4)	5 (2.7)	0.79
Endocrinology	5 (1)	3 (1)	0 (0)	2 (1)	1
ENT	1 (< 1)	0 (0)	1 (1)	0	–
Gastrointestinal	6 (1)	3 (1)	0 (0)	3 (2)	1
Ingestion	16 (3)	8 (3)	0 (0)	8 (4)	1
Oncology	4 (1)	3 (1)	0 (0)	1 (1)	0.65
Nephrology	1 (< 1)	0 (0)	0 (0)	1 (1)	–
**Total bolus fluid volume, ml/kg**	41 (25,64)	40 (20,61)^a^	31 (20, 51)^ab^	57 (39, 72)^b^	< 0.001
**Total Cl load (mEq/L)**	154 (133,154)	154 (154,154)^a^	110 (110,110)^b^	138.5 (132,143)^c^	< 0.001
**Total Na load (mEq/L)**	154 (142, 154)	154 (154,154)^a^	130 (130,130)^b^	146 (142,148)^c^	< 0.001
**Severe Day 2–3 AKI**	41 (7.3)	20 (6.7)	9 (12.2)	12 (6.4)	0.24
**PICU LOS, days**	4 (3,8)	4 (3,7)	5 (3,8)	4 (3,8)	0.56
**28-day Mortality, n (%)**	20 (3.6)	8 (2.7)	2 (2.7)	10 (5.3)	0.29

*NaCl* sodium chloride, *LR* lactated ringers, *PRISM III* Pediatric Risk of Mortality III score, *Cl* chloride, *Na* sodium, *CNS* central nervous system, *ENT* ear nose throat, *AKI* acute kidney injury

Continuous variables are reported as median (IQR)

Superscripts indicate significantly different groups on pairwise comparisons after Bonferroni correction

Compared to patients enrolled from CHCO, patients enrolled at CCHMC were older (Supplemental Table 1). While there was no significant difference in total fluid bolus volume received across groups, the distribution of patients receiving mixed fluid and 0.9%NaCl at CCHMC were nearly equal, whereas patients from CHCO most often received 0.9%NaCl. There were no significant differences in PICU LOS, day 2–3 severe AKI, or 28-day mortality between the two sites.

#### Volume of fluid administration by fluid bolus exposure group

Table 1 summarizes the volume of fluid boluses received indexed for weight and the resultant sodium and chloride loads administered for each fluid exposure group. Only fluid administered as “bolus” was included in the total bolus volume calculation. Among the 559 patients in the cohort, the median volume of fluid boluses administered was 41 [IQR 25, 64] ml/kg. There was no difference in the volume of fluid given to the 0.9%NaCl group compared to the LR group, but the mixed group received a greater volume (median of 57 [IQR 39, 72] ml/kg) in fluid boluses (*p* < 0.001). The mixed group typically received at least one bolus of 0.9%NaCl and at least one bolus of LR. Sodium and chloride load was also different across fluid exposure groups, with the highest load of each seen in the 0.9% NaCl group, and the lowest in the LR group.

#### Electrolyte values by fluid exposure group

**Fig. 1:**
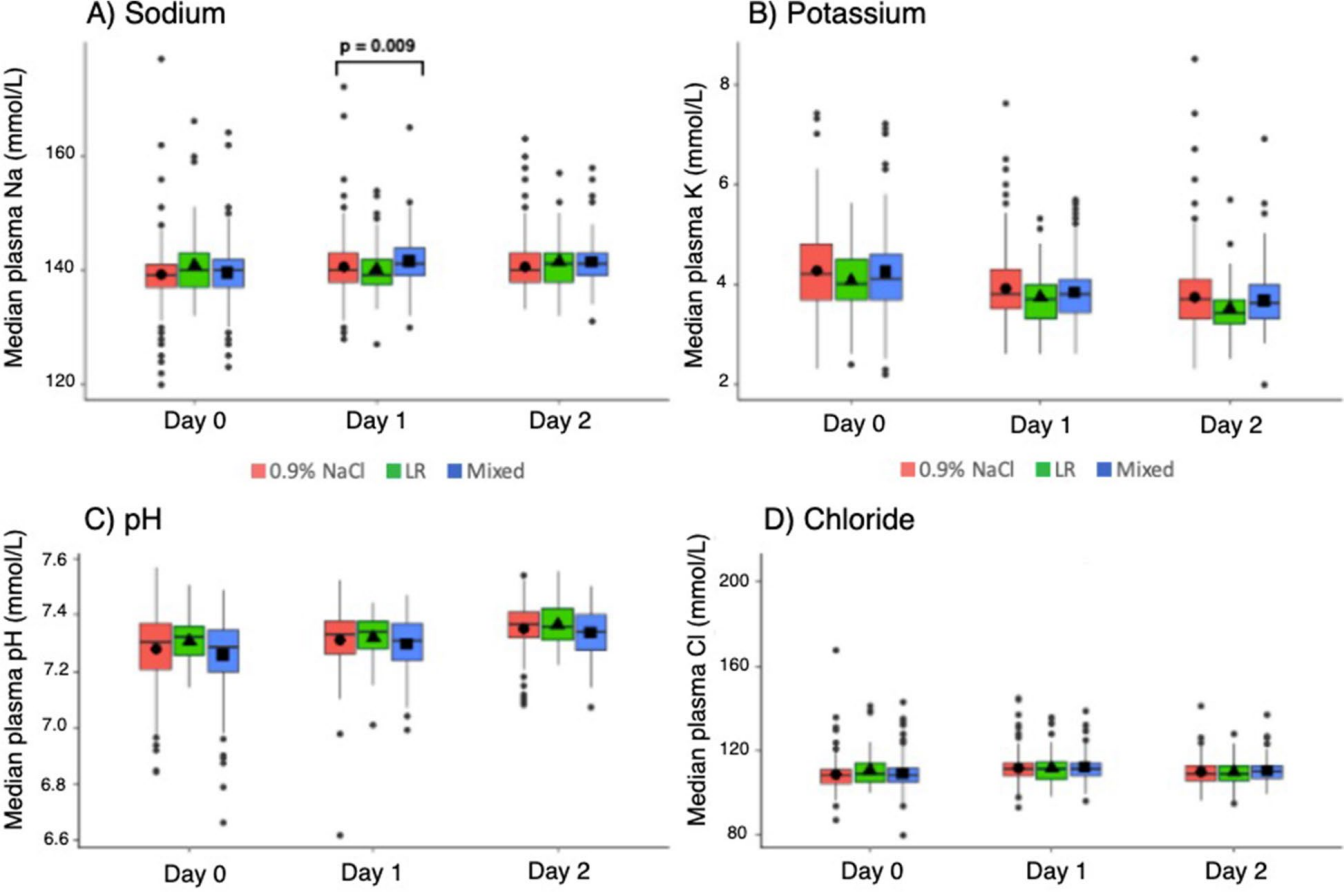
Comparison of median sodium (**A**), potassium (**B**), pH (**C**) and chloride (**D**) values on Day_0_ through Day_2_ of admission by fluid bolus exposure group. Error bars indicate [Q1, Q3]

Table 2 summarizes the median daily sodium, potassium, chloride, and pH values for each fluid exposure group from Day_0_ to Day_2_. There were no significant differences in measured electrolytes or pH between the three groups on Day_0_. Median sodium values differed significantly across groups on Day_1_, with highest values seen in the mixed group (median 141 [IQR 139,143]) and lowest in the LR group (median 139 [IQR 138,142]) (*p* = 0.009). There were no other significant differences in median sodium, potassium, pH, or chloride values on Day_1_ or Day_2_, although the median potassium values were noted to be highest each day in the 0.9%NaCl group and lowest in the LR group (Fig. 1).

**Table 2:** Comparison of daily electrolyte values from Day_0_ to Day_2_ of PICU admission by fluid bolus exposure groups

	**Overall**	**0.9%NaCl**	**LR**	**Mixed**	*p*-value*
Sodium (mmol/L)					
Day_0_	140 (137, 142)	139 (137, 141)	140 (137, 143)	140 (137, 142)	0.45
Day_1_	140 (138, 143)	140 (138, 143)^ab^	139 (138, 142)^a^	141 (139, 144)^b^	0.009
Day_2_	141 (138, 143)	140 (138, 143)	141 (138, 143)	141 (139, 143)	0.03
Potassium (mmol/L)					
Day_0_	4.1 (3.7, 4.7)	4.2 (3.7, 4.8)	4.0 (3.7, 4.5)	4.1 (3.7, 4.6)	0.33
Day_1_	3.8 (3.4, 4.2)	3.8 (3.5, 4.3)	3.7 (3.3, 4.0)	3.8 (3.4, 4.1)	0.15
Day_2_	3.6 (3.3, 4.0)	3.7 (3.3, 4.1)	3.4 (3.2, 3.7)	3.6 (3.3, 4.0)	0.06
pH					
Day_0_	7.30 (7.21,7.36)	7.30 (7.21,7.37)	7.32 (7.26,7.36)	7.28 (7.20,7.35)	0.09
Day_1_	7.32 (7.25,7.38)	7.33 (7.26,7.38)	7.34 (7.28,7.38)	7.31 (7.24,7.37)	0.20
Day_2_	7.35 (7.30,7.41)	7.37 (7.32,7.41)	7.36 (7.31,7.42)	7.34 (7.28,7.40)	0.21
Chloride (mmol/L)					
Day_0_	108 (105, 112)	108 (105, 111)	109 (105, 114)	108 (105, 112)	0.27
Day_1_	111 (108, 114)	111 (108, 114)	111 (107, 115)	111 (108, 114)	0.88
Day_2_	109 (106, 113)	109 (106, 113)	109 (106, 113)	1 10(107, 113)	0.69

*NaCl* sodium chloride, *LR* lactated ringers

All continuous values reported as median (IQR)

Superscripts indicate significantly different groups on pairwise comparisons after Bonferroni correction

^*^p-values come from Kruskal Wallis Test; significance level set at *p* < 0.017 to adjust for multiple testing per electrolyte

Episodes of laboratory-defined and clinically significant hyponatremia, hyperkalemia, hyperchloremia and acidosis are summarized in Supplemental Table 2. There were no significant differences between the groups in the rates of laboratory-defined hyponatremia, hyperkalemia, hyperchloremia, or acidosis.

#### Incidence of extreme electrolyte derangements by fluid bolus exposure group

**Fig. 2:**
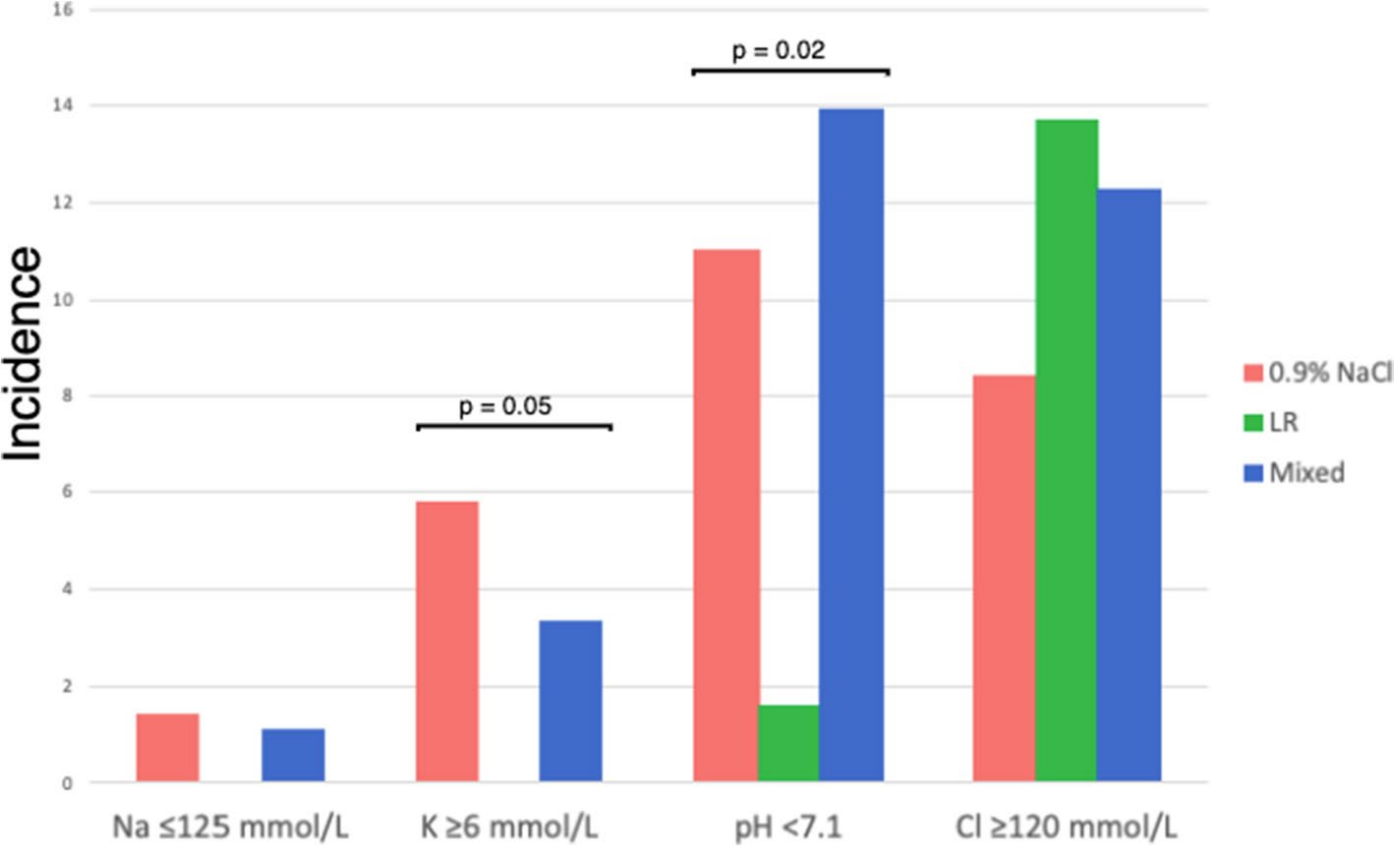
Incidence of extreme electrolyte anomalies and acidosis by fluid bolus exposure group. Na- sodium, K- potassium, Cl- chloride

When more extreme cutoffs for electrolyte and acid–base disturbances were assessed, significant differences were noted (Fig. 2, Supplemental Table 2). Severe hyperkalemia (potassium ≥ 6 mmol/L) was more commonly seen in the 0.9%NaCl fluid group (5.8%) and mixed fluid group (3.3%), with no patients in the LR group meeting this threshold (*p* = 0.05). Forty-nine patients (11%) had severe acidosis (pH < 7.1), and this was most commonly observed in patients receiving mixed fluids (13.9%) and 0.9%NaCl (11%), compared to LR (1.6%) (*p* = 0.02); only 1 patient who received LR had severe acidosis during this timeframe. Conversely, there were no significant differences in rates of extreme hyperchloremia (chloride ≥ 120 mmol/L) or hyponatremia (sodium ≤ 125 mmol/L) across the three fluid groups. While only six patients (1.1% of cohort) suffered severe hyponatremia (sodium ≤ 125 mmol/L), none of these patients were in the LR group.

#### Electrolyte values by fluid bolus exposure group in patients receiving large volume resuscitation (≥ 60 ml/kg)

A sub-analysis was performed to analyze electrolyte anomalies in patients who received ≥ 60 ml/kg of bolus fluid volume (Supplemental Table 3). There were 192 patients who received more than 60 ml/kg in this analysis, or which 92 were in the 0.9%NaCl group, 14 in LR group, and 86 in mixed group. There were no significant differences in sodium values or the incidence of acidosis across the three fluid groups. Higher rates of hyperkalemia (potassium ≥ 5.5 mmol/L) were more commonly seen in the LR group (14%) and mixed fluids groups (12%), compared to the 0.9%NaCl group (3%) (*p* = 0.048); however, these differences were not observed at other potassium thresholds. Hyperchloremia was common across all groups, and significant differences in incidence were seen at cutoffs of both ≥ 110 mmol/L (*p* = 0.026) and ≥ 115 mmol/L (*p* = 0.037). At both of these cutoffs, the incidence of hyperchloremia was highest in the LR group (86% and 64%, respectively) and lowest in the 0.9%NaCl group (62% and 29%, respectively). These differences were not observed at a hyperchloremia cutoff of ≥ 120 mmol/L.

## Discussion

In this large, two-center prospective observational study, we found that resuscitation with LR was not associated with significant electrolyte abnormalities when compared to the use of 0.9%NaCl. While there were no clinically significant differences in daily median electrolyte values between patients, those who received LR had a lower incidence of extreme hyperkalemia and acidosis compared to those who received 0.9%NaCl or mixed fluids, and suffered no instances of severe hyponatremia. Taken together, the results of our study suggest that LR appears to be a safe alternative to 0.9%NaCl as a resuscitative fluid in a heterogeneous population of critically ill children. This adds to the known literature demonstrating that balanced crystalloids are associated with fewer electrolyte derangements in critically ill adults [4, 12].

Evaluating the impact of fluid selection on the rates of clinically meaningful hyperkalemia and hyponatremia in critically ill children is necessary, as a single instance of either of these events can have important clinical consequences. For instance, one episode of hyponatremia can lead to seizures and other neurologic sequelae, while an instance of severe hyperkalemia can result in cardiac dysrhythmia and/or arrest [27, 28]. In this large study, there were no episodes of extreme hyperkalemia (≥ 6 mmol/L) or hyponatremia (≤ 125 mmol/L) in patients resuscitated with predominantly LR fluids; conversely, there were episodes of extreme hyperkalemia (≥ 6 mmol/L) and hyponatremia (≤ 125 mmol/L) in those receiving 0.9%NaCl. While this provides some evidence that LR is safe for use in a heterogeneous cohort of critically ill children, the observational nature of this study makes the significance of these findings difficult to interpret. Specifically, we are unable to delineate whether the administration of 0.9%NaCl caused the aforementioned electrolyte derangements, or if a physician chose 0.9%NaCl precisely because of those findings (i.e., selecting 0.9%NaCl to resuscitate a patient with hyponatremia).

While the causative nature of these findings cannot be established, the finding that LR, which contains a minimal amount of potassium (4 mEq/L), leads to fewer instances of hyperkalemia than 0.9%NaCl (which contains no potassium) does have a physiologic basis. First, administering LR to a patient with hyperkalemia will dilute the amount of extracellular potassium through a weighted average. For example, a 15 kg patient has a total body water estimated at 10 L, of which one third is extracellular (3.25 L). If this patient has an initial potassium level of 5 mmol/L and receives 60 ml/kg of fluid resuscitation with LR, the extracellular fluid compartment will increase to 4.15 L. In this scenario, the final serum potassium level would actually *decrease*to 4.78 mmol/L, due to the relatively greater expansion of the extracellular fluid compartment compared to potassium load. Additionally, most of the body’s potassium is intracellular, and thus any potassium shifting into the extracellular compartment will lead to an increased serum potassium level. Since the pH of 0.9%NaCl is 5, it can induce a metabolic acidosis [29–32], resulting in intracellular shifts of hydrogen ions in exchange for potassium, which increases extracellular potassium levels [33]. This effect has been demonstrated previously in kidney transplant recipients, where patients who received 0.9%NaCl had higher rates of acidosis and hyperkalemia compared to those who received either LR [3, 34, 35] or PL [13, 36]. Our study adds to this body of evidence, as severe acidosis (pH < 7.1) was indeed seen more commonly in patients who received both 0.9%NaCl and mixed fluids, and these same groups also had a higher incidence of severe hyperkalemia (potassium ≥ 6 mmol/L) compared to those receiving LR. While it is unclear the reasons these patients developed severe acidosis it is likely multifactorial and includes both the significantly higher volume compared to LR fluids and/or severity of illness.

This study has important limitations. First, this was an observational study, and thus the type of fluid administered was based on the clinical judgement of the physician and not assigned at random. Additionally, practice variations suggested by the subgroup analysis in resuscitative fluid selection at the two study sites may have resulted in bias, although there were relatively equal numbers of patients included from each center. Fluid selection also differed based on admission diagnosis. Notably, our database did not reliably capture the timing of electrolyte measurement as compared to fluid administration on Day_0_, and thus there is the potential that the type of fluid bolus chosen was based on the admission electrolyte values. Additionally, we were unable to capture other sources of fluid intake such as medication volume, although were able to include both maintenance and bolus fluids in our analysis. Maintenance and bolus fluids were considered similarly due to inconsistent charting in the EHR that often made it difficult to consistently determine whether a fluid was “bolus” or “maintenance”. However, this allowed us to capture a more robust image of the fluid exposure given to these patients.

The interpretation of the non-parametric approach (Kruskal–Wallis test) to comparing daily electrolyte value may have indicated group differences in distributions; however, given there were no clinically significant differences in median Day_0_ sodium, chloride, potassium or pH values between groups, it seems unlikely that this led to significant bias. Finally, the type of acidosis was not determined, and respiratory acidosis may have contributed to the lower pH seen in our analysis.

Our study also has several important strengths. This is the first study to examine differences in resultant electrolytes in a heterogeneous population of critically ill children receiving resuscitation with balanced versus unbalanced crystalloids. Additionally, given inclusion of patients from two large quaternary care hospitals, it is likely that these findings may be more generalizable across the general PICU population.

## Conclusions

In a heterogeneous population of critically ill children undergoing fluid resuscitation, the incidence of significant electrolyte derangements was small, and less common in patients receiving LR compared to those receiving 0.9% NaCl or mixed fluids. This study provides important information regarding resuscitative fluid safety in this unique population, and supports comparing the use of LR, 0.9% NaCl, and a mixed fluid group in a prospective interventional trial, such as is currently ongoing in pediatric sepsis [37].

## Supplementary Information

**Additional file 1.**

## Data Availability

The data that supports the findings of this study are not openly available due to them containing information that could compromise research participant privacy. Data may be available from the corresponding author upon reasonable request.
