# Supplementary material for: Electrolyte derangements in critically ill children receiving balanced versus unbalanced crystalloid fluid resuscitation
Source: BMC Nephrol. 2022 Dec 6;23:388. doi: 10.1186/s12882-022-03009-w (PMC9727874; doi:10.1186/s12882-022-03009-w)
Supplement: Supplementary file 1 — Additional file 1. [file 12882_2022_3009_MOESM1_ESM.docx]

**SUPPLEMENTARY MATERIAL:**

**Supplemental Table 1:** Basic demographic, clinical and outcome data by study site.

|  | CHCO | CCHMC | p-value |
| --- | --- | --- | --- |
| N (% cohort) | 259 | 300 | -- |
| Age, months | 30.5 (13.8,99) | 64 (16.8,155) | <0.001 |
| Gender, n (% male) | 148 (57) | 163 (54) | 0.56 |
| Weight, kg | 12.9 (9.5,23.1) | 19.8 (10,42.6) | <0.001 |
| PRISM III | 4 (0,8) | 3 (0,7) | 0.41 |
| Total bolus fluid volume, ml/kg | 41 (21,62) | 42 (29,68) | 0.65 |
| Bolus Exposure Group, n (%)  0.9%NaCl  LR  Mixed | 156 (60)  50 (19)  53 (21) | 141 (47)  24 (8)  135 (45) | 0.003  <0.001  <0.001 |
| Day 2-3 Severe AKI | 22 (8.5) | 19 (6.3) | 0.42 |
| PICU LOS, days | 4.7 (2.9,7.7) | 4 (2,8) | 0.098 |
| 28-day Mortality, n (%) | 11 (4.2) | 9 (3) | 0.57 |

CHCO- Children’s Hospital Colorado; CCHMC- Cincinnati Children’s Hospital Medical Center; 0.9%NaCl- sodium chloride; LR- lactated ringers; PRISM III- Pediatric Risk of Mortality III score; Cl- chloride; Na- sodium; AKI- acute kidney injury

Continuous variables are reported as median (IQR)

Superscripts indicate significantly different groups on pairwise comparisons after Bonferroni correction.

**Supplemental Table 2.** Comparison of the incidence of electrolyte derangements from Day_0_ to Day_2_ of PICU admission by fluid bolus exposure group.

|  | Overall | 0.9%NaCl | LR | Mixed | p-value |
| --- | --- | --- | --- | --- | --- |
| Sodium ≤ 125 mmol/L, n (%) | 6 (1.1) | 4(1.4) | 0 (0) | 2 (1.1) | 1 |
| Sodium ≤ 130 mmol/L, n (%) | 23 (4.1) | 13 (4.4) | 1 (1.4) | 9 (4.8) | 0.50 |
| Sodium ≤ 135 mmol/L, n (%)* | 109 (19.6) | 55 (18.6) | 16 (21.9) | 38 (20.3) | 0.79 |
| Potassium ≥5 mmol/L, n (%)* | 115 (20.9) | 67 (22.9) | 11 (15.1) | 37 (20.1) | 0.34 |
| Potassium ≥5.5 mmol/L, n (%) | 51 (9.3) | 29 (9.9) | 2 (2.7) | 20 (10.9) | 0.09 |
| Potassium ≥6.0 mmol/L, n (%) | 23 (4.2) | 17 (5.8) | 0 (0) | 6 (3.3) | 0.05 |
| Chloride ≥110 mmol/L, n (%)* | 406 (73) | 210 (70.9) | 48 (65.8) | 148 (79.1) | 0.05 |
| Chloride ≥115 mmol/L, n (%) | 174 (31.3) | 84 (28.4) | 26 (35.6) | 64 (34.2) | 0.28 |
| Chloride ≥120 mmol/L, n (%) | 58 ( 10.4) | 25 (8.4) | 10 (13.7) | 23 (12.3) | 0.22 |
| pH <7.3, n (%)* | 277 (61.0) | 128 (56.4) | 39 (63.9) | 110 (66.3) | 0.125 |
| pH <7.2, n (%) | 117 (25.8) | 57 (25.1) | 10 (16.4) | 50 (30.1) | 0.106 |
| pH <7.1, n (%) | 49 (10.8) | 25 (11.0)^ab^ | 1 ( 1.6)^a^ | 23 (13.9)^b^ | 0.016 |

NaCl- sodium chloride; LR- lactated ringers

Superscripts indicate significantly different groups on pairwise comparisons after Bonferroni correction.

*Laboratory-defined cutoff value

**Supplemental Table 3.** Incidence of electrolyte derangements by fluid bolus exposure group for subgroup of patients receiving more than 60 ml/kg in bolus fluid.

|  | Overall  (n=192) | 0.9%NaCl  (n=92) | | LR  (n=14) | Mixed  (n=86) | p-value |
| --- | --- | --- | --- | --- | --- | --- |
| Day 0-2 Sodium ≤125 mmol/L, n (%) | 1 (0.5) | 1 (1.1) | 0 (0) | | 0 (0) | 1.0 |
| Day 0-2 Sodium ≤130 mmol/L, n (%) | 9 (4.7) | 5 (5.5) | 0 (0) | | 4 (4.7) | 1.0 |
| Day 0-2 Sodium ≤135* mmol/L, n (%) | 48 (25) | 23 (25) | 3 (21) | | 22 (26) | 1.0 |
| Day 0-2 Potassium ≥5* mmol/L, n (%) | 36 (19) | 15 (17) | 6 (43) | | 15 (18) | 0.078 |
| Day 0-2 Potassium ≥5.5 mmol/L, n (%) | 15 (7.9) | 3 (3.3)^a^ | 2 (14)^ab^ | | 10 (12)^b^ | 0.048 |
| Day 0-2 Potassium ≥6.0 mmol/L, n (%) | 5 (2.6) | 1 (1.1) | 0 (0) | | 4 (4.7) | 0.35 |
| Day 0-2 Chloride ≥110* mmol/L, n (%) | 136 (71) | 57 (62)^a^ | 12 (86)^b^ | | 67 (79)^ab^ | 0.026 |
| Day 0-2 Chloride ≥115 mmol/L, n (%) | 62 (33) | 27 (29)^a^ | 9 (64)^b^ | | 26 (31)^ab^ | 0.037 |
| Day 0-2 Chloride ≥120 mmol/L, n (%) | 22 (12) | 8 (8.7) | 3 (21) | | 11 (13) | 0.27 |
| Day 0-2 pH <7.3, n (%)* | 129 (74) | 61 (73) | 11 (92) | | 57 (72) | 0.39 |
| Day 0-2 pH <7.2, n (%) | 61 (35) | 30 (36) | 4 (33) | | 27 (34) | 0.97 |
| Day 0-2 pH <7.1, n (%) | 24 (14) | 15 (18) | 0 (0) | | 9 (11) | 0.22 |

NaCl- sodium chloride; LR- lactated ringers; AKI- acute kidney injury

Continuous variables are reported as median (IQR)

Superscripts indicate significantly different groups on pairwise comparisons after Bonferroni correction.

*Laboratory-defined cutoff value
